# Lunar Cycle Influences Spontaneous Delivery in Cows

**DOI:** 10.1371/journal.pone.0161735

**Published:** 2016-08-31

**Authors:** Tomohiro Yonezawa, Mona Uchida, Michiko Tomioka, Naoaki Matsuki

**Affiliations:** 1 Department of Veterinary Medical Sciences, The University of Tokyo, Tokyo, Japan; 2 School of Veterinary Medicine, Kitasato University, Aomori, Japan; University of Texas Southwestern Medical Center, UNITED STATES

## Abstract

There is a popular belief that the lunar cycle influences spontaneous delivery in both humans and cattle. To assess this relationship, we investigated the synodic distribution of spontaneous deliveries in domestic Holstein cows. We used retrospective data from 428 spontaneous, full-term deliveries within a three-year period derived from the calving records of a private farm in Hokkaido, Japan. Spontaneous birth frequency increased uniformly from the new moon to the full moon phase and decreased until the waning crescent phase. There was a statistically significant peak between the waxing gibbous and full moon phases compared with those between the last quarter and the waning crescent. These changes were clearly observed in deliveries among multiparous cows, whereas they were not evident in deliveries among nulliparous cows. These data suggest the utility of dairy cows as models for bio-meteorological studies, and indicate that monitoring lunar phases may facilitate comprehensive understanding of parturition.

## Introduction

In hospital delivery units, there is frequently a perception that the onset of delivery is influenced by environmental factors. Climatic conditions, such as atmospheric pressure, time of day, and weekly patterns, are reported to influence admission for labor and/or delivery [1–3]. It is also believed that the phase of the lunar cycle affects delivery timing; however, most of the scientific reports concerning lunar influence on deliveries remain controversial [3–13].

Although epidemiological research is the best approach for direct observation of human physiological phenomena, it is sometimes difficult to draw conclusions because differences in genetic, nutritional, environmental, and social backgrounds can cause complex variations in the data. Thus, to minimize the influence of such factors, we focused on data from domestic Holstein cows. It is known that domestication generally decreases the genetic diversity of strains and that the Holstein breed has much lower genetic variability than other cattle breeds based on microsatellite DNA analysis [14]. In addition, they are rigorously managed under uniform conditions by professional dairy farmers. In the cattle industry, it is likewise believed that the full moon influences delivery. Weather patterns, such as changes in atmospheric pressure and temperature, have been reported to affect timing of birth in cattle [15], which is consistent with comparable reports for humans [1–3]. Hence, in the present study, we investigated the relationship between lunar phases and calving dates of Holstein cows.

## Materials and Methods

We used the calving records from one private farm, which belonged to the National Agricultural Insurance Association (NOSAI) in Ishikari (Hokkaido, Japan), for the three-year period between September 1, 2011 and August 31, 2013. There were always around 300 adult, 50 growing cattle and 30 calves in the farm. The commercial purpose of the farm was dairy husbandry. We included 428 spontaneous full-term deliveries that were not driven by any parturifacients (S1 Table). The mean age of the 428 Holstein cows was 3.52 (SD = 1.62) years and the mean gestational age was 283.54 (SD = 4.04) days. Thus, the predicted delivery dates were defined as 284 days after the dates of final artificial insemination (AI) in this study. Among 428 deliveries, 145 were from nulliparous heifers (33.9%) and 283 (66.1%) were from multiparous cows. These 428 deliveries were obtained from 314 individual cows; 200 cows delivered once and 114 cows delivered twice during the observation period. Out of 114 cows, 43 experienced their first and second deliveries, 44 their second and third, 12 their third and fourth, six their fourth and fifth, five their fifth and sixth, two their sixth and seventh, and two their seventh and eighth.

The length of the estrous cycle in cows is approximately 21 days and the timing of AI was decided using the a.m.-p.m. rule by the local veterinarian. Cows were pastured or reared in a free stall (10 m^2^/head) until approximately 60 days before the predicted delivery date and were then reared in a free barn (10 m^2^/head) until delivery. Free stalls had individual spaces for cows and they were loosely restrained within these. Free barns did not have the equipment to restrain cattle and cows were put within the compartment surrounding the fences. Free stalls and barns did not have night illumination or robust walls. The delivery date was defined as 6:00 a.m. on the day parturition was completed until 6:00 a.m. the next morning.

The exact timing of the lunar phases and weather reports were derived from data presented by the Japanese Meteorological Agency. Since the length of a lunar month is 29.53 days, the eight phases of the moon were categorized; new moon to waxing crescent (0 to 3.69), wax crescent to first quarter (3.69–7.38), first quarter to waxing gibbous (7.38–11.07), waxing gibbous to full moon (11.07–14.76), full moon to waning gibbous (14.76-–18.45), waning gibbous to last quarter (18.45–22.14), last quarter to waning crescent (22.14–25.83), and waning crescent to new moon (25.83–29.53). The birth frequency was defined as the number of births in the lunar phase in relation to all deliveries.

Statistical analyses were performed as reported by Ochiai *et al*. [5] using R based software (ver. 3.2.2, R Foundation for Statistical Computing, Vienna, Austria). Distributions of deliveries during the lunar phases were compared using χ^2^ tests followed by multiple comparisons using Ryan-Holm step-down Bonferroni tests. Differences between the actual and predicted delivery dates were compared using Kruskal-Wallis tests and Steel-Dwass post hoc tests. Differences were considered significant when *p* < 0.05.

## Results

**Fig 1:**
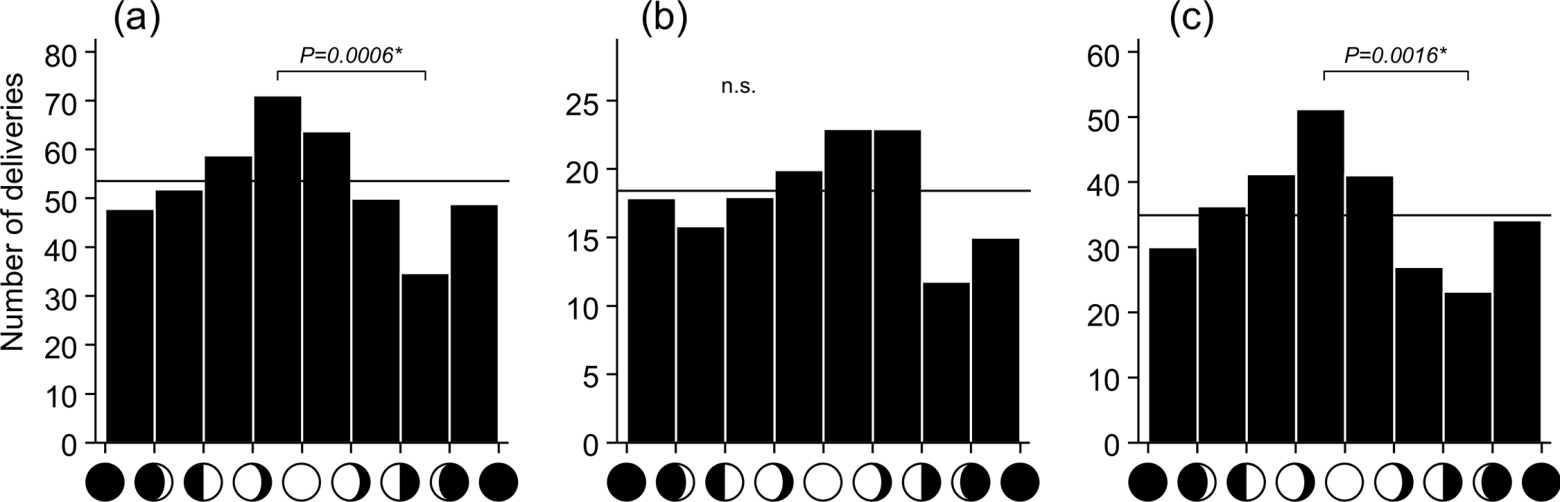
Distribution of spontaneous deliveries according to the lunar cycle. *P* values indicate significant differences in multi-comparison tests; n.s., not significant. Expected number of deliveries is indicated using horizontal lines: (a) total deliveries, (b) nulliparous deliveries, (c) multiparous deliveries.

Among all spontaneous deliveries, birth frequency increased uniformly from the new moon to the full moon phase, and then decreased until the waning crescent (Fig 1A). The results of the χ^2^ test (χ^2^ = 15.963, d.f. = 7, *p* = 0.0255) warranted rejection of the null hypothesis, and subsequent multiple comparisons indicated that the number of deliveries was significantly higher between the waxing gibbous and full moon phases (*p* = 0.0006).

Fig 1B and 1C show the distribution of deliveries among nulliparous and multiparous cows according to the lunar cycle. Delivery timing among nulliparous cows did not significantly change throughout the lunar cycle, although it exhibited a tendency to increase between the waxing gibbous and full moon phases (χ^2^ = 5.68, d.f. = 7, *p* = 0.5781, Fig 1B). In contrast, there was a clear and significant relationship between delivery timing among multiparous cows and the lunar cycle (χ^2^ = 15.88, d.f. = 7, *p* = 0.0262, Fig 1C). Subsequent multiple comparison tests showed that the number of deliveries was significantly greater between the waxing gibbous and full moon phases compared with that between the last quarter and the waning crescent phases (*p* = 0.0016).

**Fig 2:**
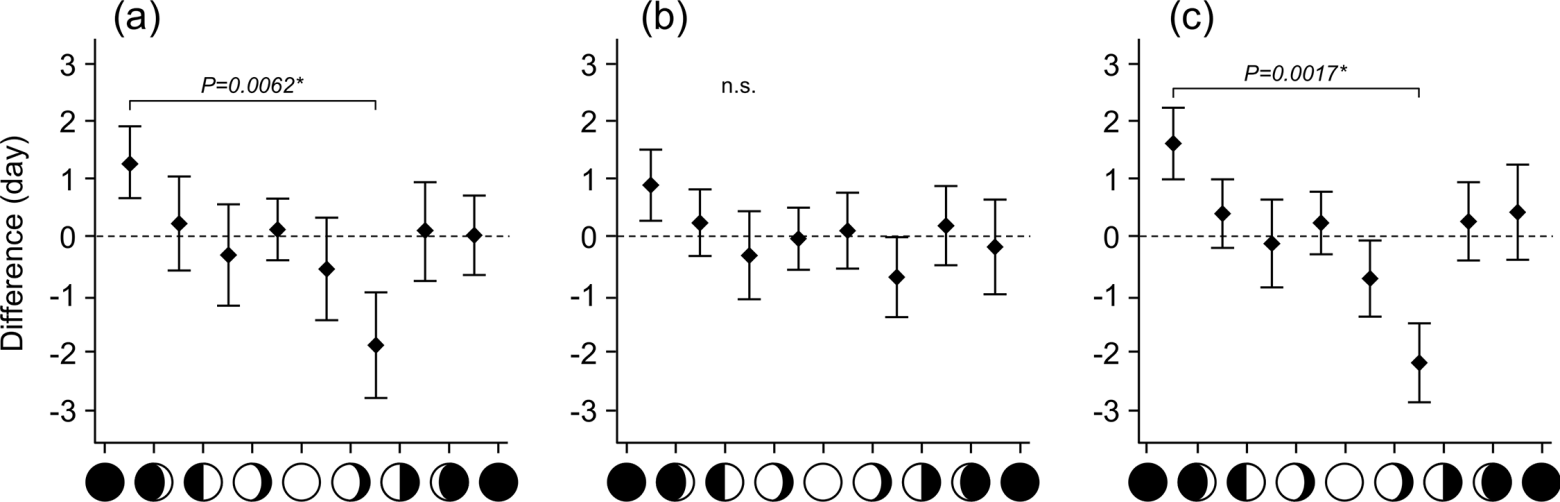
Differences between actual and predicted delivery dates. Predicted delivery dates were calculated to be 284 days after the date of the final AI. The difference in the number of days between actual and predicted delivery dates (gaps) were plotted based on the lunar phase of the predicted dates. Squares and bars indicate means and SEMs for each lunar phase. (a) total deliveries, (b) nulliparous deliveries, (c) multiparous deliveries.

In further analyses, the numbers of days between predicted and actual delivery dates differed significantly among the eight moon phases for all cows, and for multiparous cows (Fig 2). Fig 2 presents an analysis based on the same parturition data used in Fig 1. Specifically, cows with predicted delivery dates during the new moon to first quarter phase tended to delay delivery, whereas those with predicted delivery dates during the full moon to last quarter phase tended to deliver when predicted. Finally, a significant difference was observed between the new moon to waxing crescent phase and the waning crescent to last quarter phase (Fig 2A and 2C).

To determine whether the difference in delivery dates could be caused by individual specificity, the correlation of gaps exhibited by the same cows was analyzed. It is visualized as a scattered plot in S1 Fig. This figure shows the correlation of gaps between two deliveries for each cow (n = 114). There were no correlations between the gaps for the former and latter delivery dates for the same cows (R^2^ = 0.1162, *p* = 0.5925). This suggested that the difference between the actual and predicted delivery date was not related to individual specificity.

**Fig 3:**
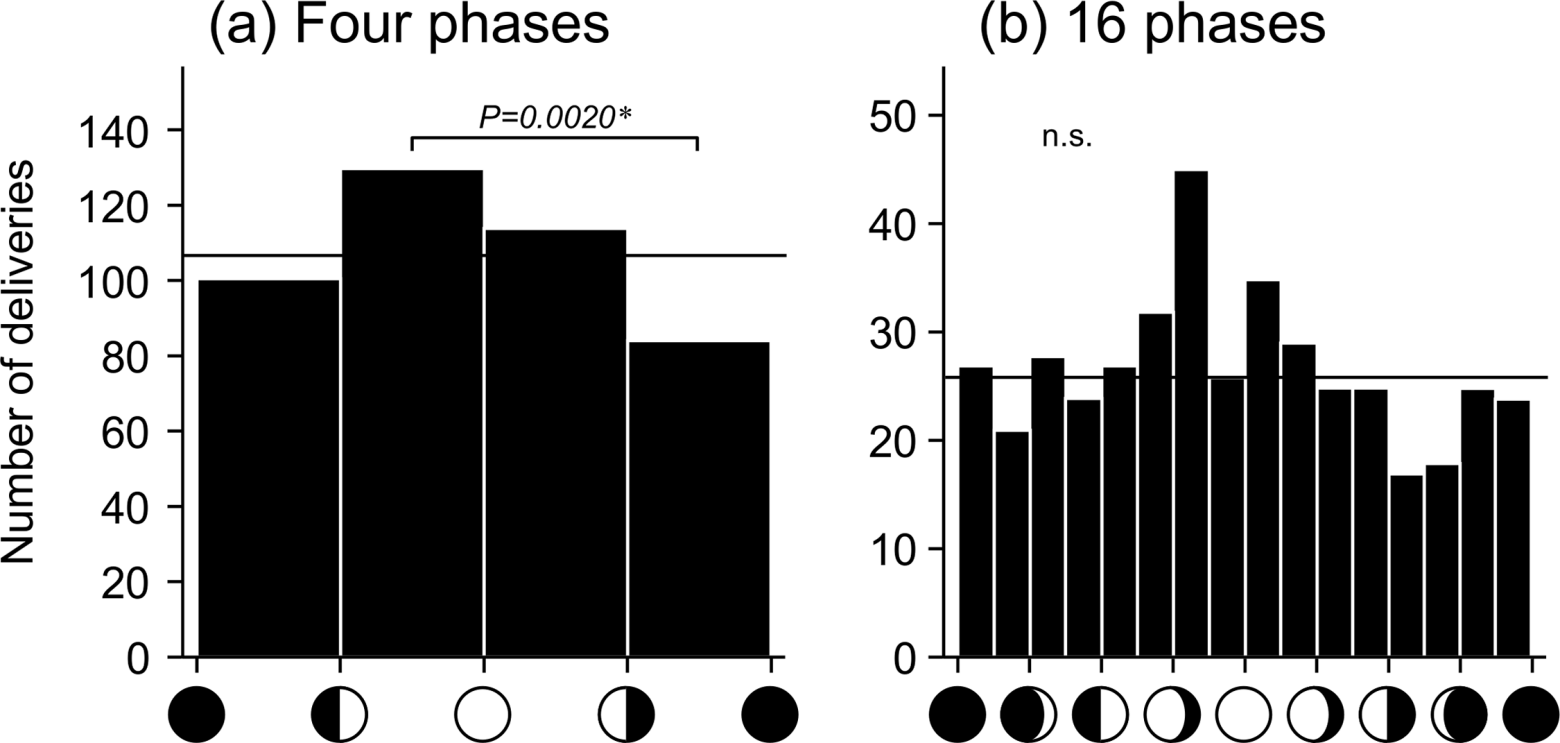
Broad and detailed distribution of spontaneous deliveries within the lunar cycle. (a) The lunar cycle separated into four phases (*χ*^2^ = 10.88, d.f. = 3, *p* = 0.0124). (b) The lunar cycle separated into 16 phases (*χ*^2^ = 24.86, d.f. = 15, *p* = 0.0519). *P* values indicate significant differences based on multiple-comparison tests; n.s., not significant; expected numbers of deliveries are indicated by horizontal lines.

To assess data quality, we separated the lunar cycle into broad and detailed phases using the same data. When the lunar cycle was separated into four phases, a significant alteration was observed (Fig 3A). However, when it was separated into 16 phases, no significant difference was observed, although a peak in deliveries was observed around the full moon phase (Fig 3B).

In addition, correlation between delivery date and weather was analyzed. According to weather reports, there were 393 sunny, 305 cloudy, 18 rainy, and 15 snowy days during the observation period (Sep 2011 to Aug 2013). The weather conditions for the 428 deliveries were 237 sunny, 179 cloudy, four rainy, and eight snowy days. There was no significant relationship between weather conditions and delivery timing (χ^2^ = 3.495, d.f. = 3, *p* = 0.3215).

## Discussion

Although there has been a pervasive belief that the lunar cycle can influence labor onset and parturition, most researchers remain skeptical based on retrospective evidence. Two studies from the USA [11, 12], one from Germany [16], and one from Brazil [3] showed no lunar influence on hospital admissions for labor. Although Ghiandoni *et al*. reported a higher rate of childbirth in Italy during the full moon phase [13], they also claimed that the association was too weak to predict the days with the highest frequency of deliveries. However, various factors could influence birth frequency in humans, which could mask the influence of the lunar phase. For instance, obesity affects the length of gestation [17], weekends tend to yield reduced childbirth frequency, and there are peaks in labor admission rates on Mondays and Fridays [3]. To minimize the effects of these factors, we focused on domestic Holstein cows. Compared to humans, the subjects were more genetically related, because spermatozoa of breeding cattle were rigorously selected. Their feeding and nutrition were uniformly managed by dairy farmers, and their behavior and social interactions were not as complex as those of humans. Elimination of some of the sources of variation inherent in humans could be one of the reasons why our data showed a clear trend in comparison with that of previous reports. Nevertheless, the present results cannot be extrapolated directly to humans because reproductive physiology can be quite different between species.

Ghiandoni *et al*. reported the relationship between parity and delivery timing during the various lunar phases [13]. Although they found a clear and significant cluster of deliveries around the time of the full moon in multiparae, they adopted a cautious stance in drawing conclusions because differences could have existed between reality and memory regarding the actual time of delivery for previous pregnancies [13]. In the present study, we also found a significant increase in the number of deliveries around the full moon phase in multiparae, whereas a similar trend was not evident in nulliparous dairy cows. The insemination and parturition records of cattle are accurate in comparison with human memory. Although we used data from cattle only, our findings support the relationship between lunar phases and parity hypothesized by Ghiandoni *et al*. In addition, dystocia is a widely accepted risk during first parturition in cows, reflecting smaller sizes and weights of younger primiparous dams [18]. Dystocia can last for several days in heifers, and thus advances or delays in time of birth could be sufficient to influence our findings. Hence, these complications in spontaneous deliveries among primiparous cows may obscure the influence of the lunar phase.

It is difficult to explain the mechanism by which the moon influences birth frequency. In one supposition, lunar gravity could be linked to uterine contractions through the release of oxytocin, which has also been identified as a cardiovascular hormone. In addition, plasma levels of oxytocin are increased by increases in arterial blood pressure in rats [19]. Because blood pressure is positively correlated with gravity [20], an increase in gravitational forces during the gibbous lunar phase might be a trigger for oxytocin release and subsequent delivery. Nevertheless, although changes in gravitational forces during the lunar phase are sufficient to produce tidal forces, animal weights are miniscule compared with that of the planet, and changes in gravity between lunar phases are approximately one of 0.3 millionth. Moreover, although lunar gravity increases in both the full moon and new moon phases, the birth frequency in the present study showed a significant increase only during the full moon phase. Hence, gravitational forces are an unlikely factor in the chronobiology of delivery timing.

Another explanation could be attributed to melatonin secretion. The influence of variations in lunar light on melatonin secretion has been reported; nocturnal secretion of melatonin shows a considerable decrease around the full moon phase [21]. Melatonin secretion is also increased toward the end of gestation and rapidly drops to non-pregnancy levels at parturition in humans [22]. In rats, low gravity can have a direct and/or indirect influence on delivery by altering melatonin levels [23]. In this study, there were no robust walls or artificial lights around the free stall or barns. Although we did not measure the light intensities in these areas, both the free stall and free barn were easily penetrated by moon light. Currently, humans are perpetually exposed to significant artificial lighting. Based on our hypothesis, the artificial light period could cancel the influence of the lunar cycle in humans, which might be one of the reasons why human data did not vary with the lunar phases [3–13, 16]. Thus, birth frequency might be enhanced by reduced melatonin secretion around the time of the full moon.

Because melatonin levels are closely dependent on photoperiod, it is possible that the weather condition could influence experimental data. Nevertheless, we think the effect of weather would be nullified if the experiment was performed throughout a substantial number of lunar cycles. Indeed, a previous report on humans that showed clear relationship between melatonin levels and the lunar phases did not specify the relationship of weather conditions [21]. Because they studied 64 specimens over four years, the variation in weather conditions should have been negated [21]. In this study, our data was collected throughout 25 lunar cycles. The Ishikari area of Hokkaido in Japan does not have a rainy season or experience much snow. When we analyzed the effect of weather conditions on delivery timing, no significant relationship was found. This suggests that the variation in weather conditions among the lunar phases was also negated in our study.

Another possibility is that the lunar cycle could influence the ovulation ratio and/or fertilization ratio. There are few reports based on the relationship between the lunar cycle and ovulation that can be analyzed epidemiologically. In a study that analyzed data from over 300 women, no statistical relationship was observed among them, although women tended to ovulate in the dark phase of the lunar period [24]. In the present study, there was no significant correlation between the date of AI and the lunar cycle (S2 Fig). Nevertheless, the number of AI tended to increase in the dark phase of the lunar period, which implies the same tendency as observed in the study on women [24]. However, it would be difficult to perform a detailed evaluation because data on the incidence rate of estrus, total number of AIs performed, and success rate of AIs were not collected in the present study. Nevertheless, any physiological explanations are speculative and further studies are needed to go beyond phenomenological analysis.

In the present study, we separated the lunar cycle into eight phases. When the lunar cycle was separated into four phases, there was a significant peak between the first quarter and full moon phases. However, when the cycle was separated into 16 phases, there was no significant difference, even though a peak was observed. This variation could be attributed to the relatively small sample size. It was difficult to make draw definitive conclusions because of the limited sample size in this study.

In summary, the present study used dairy cows to answer practical questions about full moon deliveries. A significant relationship between the distribution of spontaneous deliveries and the lunar phase was found, particularly among multiparous dairy cows. These results suggest that the dairy cow may be a good model for bio-meteorological study, and that monitoring lunar phases provides considerable information for understanding the influences on delivery more comprehensively.

## Supporting Information

S1 Fig Correlation between gaps of delivery dates in the same cattle. Differences between actual and predicted delivery dates in the former parturition (x axis) and the latter parturition (y axis) of each cow were plotted (n = 114). Small circles indicate n = 1. Double circles indicate n = 2. Big circles and numbers indicate overlapping sample size.
(EPS)

S2 Fig Distribution of successful AI dates according to the lunar cycle. Expected numbers of deliveries are indicated with horizontal lines; (a) Total deliveries, (b) nulliparous deliveries, and (c) multiparous deliveries. There were not statistically affected (n.s.).
(EPS)

S1 Table The raw data in this study; last AI, predicted and actual delivery date, and geophysical data (n = 428). (XLS)
